# Supplementary figures and images for: Expression of Apoptosis-Related Biomarkers in Inflamed Nasal Sinus Epithelium of Patients with Chronic Rhinosinusitis with Nasal Polyps (CRSwNP)—Evaluation at mRNA and miRNA Levels
Source: Biomedicines. 2022 Jun 13;10(6):1400. doi: 10.3390/biomedicines10061400 (PMC9220377; doi:10.3390/biomedicines10061400)

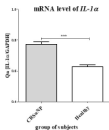

(a)

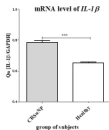

(b)

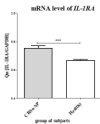

(c)

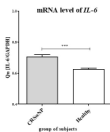

(d)

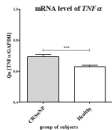

(e)

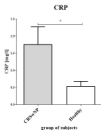

(f)

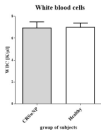

(g)

Supplement: Supplementary file 1 [file biomedicines-10-01400-s001.zip › Supplements/Figure S1.pdf]
